# New vaccines for the prevention of pneumococcal infections.

**DOI:** 10.3201/eid0204.960404

**Published:** 1996

**Authors:** H Käyhty, J Eskola

**Affiliations:** Department of Vaccines, National Public Health Institute, Helsinki, Finland. helena.kayhty@ktl.fi

## Abstract

Streptococcus pneumoniae is a major cause of acute otitis media, pneumonia, bacteremia, and meningitis. Because in recent years antibiotic-resistant pneumococcal strains have been emerging throughout the world, vaccination against pneumococcal infections has become more urgent. The capsular polysaccharide vaccine that has been available is neither immunogenic nor protective in young children and other immunocompromised patients. Several pneumococcal proteins have been proposed as candidate vaccines, but no human studies associated with them have been reported. Clinical trials of first-generation pneumococcal conjugate vaccines have shown that covalent coupling of pneumococcal capsular polysaccharides to protein carriers improves the immunogenicity of the polysaccharides. The protective efficacy of the conjugate vaccines against carriage, acute otitis media, and invasive infections is being studied.


### New Vaccines for the Prevention of Pneumococcal Infections

**Helena Käyhty and Juhani Eskola**
National Public Health Institute, Helsinki, Finland

*Streptococcus pneumoniae* is a major cause of acute otitis media, pneumonia, bacteremia, and meningitis. Because in recent years antibiotic-resistant pneumococcal strains have been emerging throughout the world, vaccination against pneumococcal infections has become more urgent. The capsular polysaccharide vaccine that has been available is neither immunogenic nor protective in young children and other immunocompromised patients. Several pneumococcal proteins have been proposed as candidate vaccines, but no human studies associated with them have been reported. Clinical trials of first-generation pneumococcal conjugate vaccines have shown that covalent coupling of pneumococcal capsular polysaccharides to protein carriers improves the immunogenicity of the polysaccharides. The protective efficacy of the conjugate vaccines against carriage, acute otitis media, and invasive infections is being studied.

*Streptococcus pneumoniae* (pneumococcus [Pnc]) is a common bacterial agent found in mild mucosal as well as severe systemic infections. Local infections, such as acute otitis media, are rather common; every child has at least one during the first 2 years of life (1), and Pnc is the causative agent in approximately half of the bacterial culture-positive cases (2). Pneumonia is another disease often caused by Pnc, both in industrialized and developing countries. Pneumonia, which causes more than one million deaths per year, is the most common cause of childhood death in the developing world (3); pneumococcal pneumonia is a serious problem among the elderly in industrialized countries. Pnc also causes frequent invasive infections, especially among children. In Finland, the incidence of bacteremic pneumococcal infections at 0 to 4 years of age has been 24.2 per 100,000 per year (4). The corresponding rate was 42 per 100,000 per year in Israel (5) and 66 per 100,000 per year in the United States (6). In addition to the young and the elderly, some of the other groups at increased risk for Pnc infection are patients with chronic cardiac or pulmonary diseases, immunocompromised patients, and especially persons with functional or anatomic asplenia (7).

Address for correspondence: Helena Käyhty, Laboratory of Vaccine Immunology, Department of Vaccines, National Public Health Institute, Mannerheimintie 166, FIN-00300 Helsinki, Finland; fax: 358-9-4744238; e-mail: helena.kayhty@ktl.fi.

The treatment of recently emerged Pnc strains that are resistant to penicillin and other antibiotics (8) is becoming a challenge. Because of the high rates of illness and death associated with pneumococcal infections and the rapidly increasing resistance of organisms that cause these infections to antimicrobial drugs, development and use of effective pneumococcal vaccines is of high priority. The progress has been rapid; in addition to polysaccharide(PS)-protein conjugate vaccines, vaccines containing pneumococcal proteins are also being developed.

### Pneumococcal Capsular Polysaccharide Vaccine

Pnc can be divided into at least 90 serotypes according to the structure of the PS in the capsule surrounding the bacterium. The capsule seems to be the most important virulence factor; all strains isolated from infections are encapsulated. The capsule helps the bacterium escape the host defense mechanisms. However, only a small fraction of all capsular types are common causes of pneumococcal infections. The list of the most common groups/types (4, 6, 7, 9, 14, 18, 19, and 23) that cause childhood infections is similar in most parts of the world. Types 1 and 5 are, however, more common in the developing world than in industrialized countries (9).

Antibodies to capsular PSs protect from infection by opsonizing Pnc for phagocytosis by neutrophils. A capsular PS vaccine containing 23 of the most common serotypes/groups has proven protective in immunocompetent adults and in some groups at risk (7,10,11); it has also been shown to have an impact on death rates due to pneumonia in Papua New Guinea (12). Among the immunocompromised and in preventing acute otitis media, (13) its efficacy has been only marginal.

The reason for the vaccine's poor immunogenicity and its lack of efficacy in children is thought to be the nature of the PS antigen. PS antigens are type 2 T-cell independent (TI) antigens, which stimulate mature B cells without the help of T cells. In humans, the B cells of newborns do not respond to most of the PS antigens. Responsiveness develops only slowly during the first years of life. Furthermore, the TI antigens do not induce immunologic memory and the maturation of the immune response; anti-PS antibodies have low avidity and the switch from one isotype to another does not happen even after repeated immunizations. The TI antigens induce mainly IgM responses, especially in mice. However, in humans the response also contains the IgG and IgA components (14). Furthermore, the IgG response to PS antigens contains a greater proportion of IgG2 (15,16) than found in a response to protein antigens. The lack of memory has some important implications for the vaccination. Because of the rapid decline of antibodies, revaccination is often necessary (7).

### Pneumococcal Protein Vaccine Candidates

Several ways have been and are being tried to solve the problem of poor immunogenicity of pneumococcal PS vaccines in infancy. In addition to the capsule, other pneumococcal virulence factors have been considered as promising vaccine candidates or as carrier proteins in pneumococcal conjugate vaccines (see above). The prime vaccine candidates are enzymes and toxins that are excreted or released after the bacterium has autolyzed or surface proteins whose exact functions are not known. Pneumococcal proteins studied as potential vaccines include neuramididase, autolysin, pneumolysin, pneumococcal surface protein A (PspA), and pneumococcal surface adhesin A (PsaA) (17-19).

Pneumolysin is a cytolytic toxin produced by all types of Pnc. In mice, immunization with inactivated pneumolysin or recombinant pneumolysin toxoid offers at least partial protection or enhanced survival when challenged with Pnc (20,21). PspA is a surface protein present in all clinically relevant pneumococcal strains. PspAs from different pneumococcal strains vary serologically. However, many PspA antibodies cross-react with PspAs from unrelated strains. Furthermore, active immunization of mice with PspA generates protective immune response against diverse pneumococcal strains (22). Truncated PspAs, expressed as recombinant proteins, are also immunogenic in mice and can elicit cross-protection (18).

### Pneumococcal Conjugate Vaccines

Another approach to solving the poor immunogenicity of the capsular PS antigens has already moved to the clinical phase-III trials. This approach is based on the 1929 findings of Goebel and Avery (23), who showed that covalent coupling of haptens to a protein carrier improves the immunogenicity of the hapten. In this way, the anti-PS response gets T-cell dependent characters: there is development of immunologic memory and maturation of the immune response. This is seen as an increase in the antibody concentrations and the antibody affinity and as a switch in the isotype distribution after repeated immunizations. This approach has been used successfully to prepare vaccines against *Haemophilus influenzae* type b (Hib); the incidence of Hib infection has decreased drastically wherever these conjugate vaccines have been used (24).

The PS antigen in a conjugate vaccine seems to benefit at least partly from the immunologic characters of the carrier protein. The protein is presented as peptides in association with the major histocompatibility complex class II molecules on the surface of the antigen-presenting cells. This stimulates the T-helper cells, which then stimulate adjacent B cells for antibody production and maturation into memory cells. Development of immunologic memory means that the protection does not depend solely on the existing antibody concentration. Instead, the vaccinated persons can respond with a rapid, high, and effective antibody response to colonization or invasion by the respective Pnc type. Studies in Finland suggest that this indeed happens: the efficacy of an Hib conjugate vaccine, PRP-D, was more than 90% in early infancy, even though a large proportion of the infants did not have measurable antibody response after the primary course of immunization (25). A study in the United Kingdom suggests that the carriage of Hib indeed induces a high "booster type" immune response (26).

Conjugation of the PS to a protein carrier has repeatedly been shown to work with Hib; vaccines based on the same principle would also decrease the number of different infections caused by Pnc. Four vaccine manufacturers have prepared pneumococcal conjugate vaccines with basically the same approaches as the Hib conjugates (Table 1). PncOMPC vaccine contains PSs from seven serotypes conjugated to the meningococcal outer membrane protein complex (27). The PncCRM vaccine contains either oligosaccharides (OS) or PSs coupled to a nontoxic mutant diphtheria toxin CRM197. The PS-containing conjugate vaccine is at present heptavalent (28), but it is possible to add types 1 and 5 to the product intended for use in developing countries. The PncT vaccine contains eight PSs coupled to tetanus toxoid, and the PncD product contains the same PSs coupled to diphtheria toxoid (29). Besides these formulations, several other approaches have been tested in animals. These include conjugates using pneumolysoid (30), pertussis toxin (31), and salmonella protein (32) as a carrier. Recently, small peptides selected on the basis of T-cell stimulating properties have also been coupled to pneumococcal PS to form conjugate vaccines (33).

### Preclinical Testing

Before human trials, these conjugates were immunogenic and protective in animals, including mice, infant monkeys, and chinchillas (34-37). All these studies indicate that conjugate vaccines have greater immunogenicity than pneumococcal PS vaccines. Even though animal studies can tell if the conjugate vaccine is immunogenic and evokes a T-cell dependent response, the final proof of conjugate vaccines' superior immunogenicity and efficacy over PS vaccines comes only from human studies. So far no animal model can mimic human immunogenicity and efficacy studies.

Table 1. Pneumococcal conjugate vaccines in phase-II and phase-III trials

| Vaccine | Serotype | Carrier | Manufacturer |
|---|---|---|---|
| PncCRM | 4, 6B, 9V, 14, 18C, 19F, 23F | CRM197 | Wyeth-Lederle Vaccines and Pediatrics |
| PncD | 3, 4, 6B, 9V, 14, 18C, 19F, 23 | Diphtheria toxoid | Connaught Laboratories |
| PncT | 3, 4, 6B, 9V, 14, 18C, 19F, 23 | Tetanus toxoid | Pasteur Merieux Serums & Vaccins |
| PncOMPC | 4, 6B, 9V, 14, 18C, 19F, 23F | Meningo-coccal OMPC | Merck Research Laboratories |

CRM = CRM197, a nontoxic variant of diphtheria toxin; D = diphtheria toxoid; T = tetanus toxoid; OMPC = outer membrane protein complex

### Clinical Testing

Pneumococcal conjugates of all the manufacturers mentioned in Table 1 have now been tested in phase-I and phase-II studies. The first human studies were done in adults with mono- or bivalent conjugates and showed that the conjugates were at least as immunogenic as the PS vaccine. Since then, up to eight valent vaccines have been used in human studies, also among infants.

### Adults and Toddlers

To show that they are safe and immunogenic, pneumococcal conjugates were first given to small numbers of adults and toddlers. Most of the reported studies have been conducted with mono- to tetravalent vaccines. The PncOMPC studies in adults show that the conjugate vaccine was well tolerated but not more immunogenic than the PS vaccine (38,39). One possible reason might be the low dose (1µg to 5µg of each conjugate) used in these studies. Different formulations of PncCRM containing either PS or OS linked to CRM197 have been tested in adults. All were well tolerated and evoked a comparable immune response (40). This was confirmed in a study in which heptavalent OS conjugate was immunogenic (28). Results of immunizing adults with PncT or PncD conjugates have been reported in two studies; both vaccines were more immunogenic than the PS vaccine (41,42). The Finnish study with tetravalent PncT and PncD showed that these conjugates can also evoke a mucosal antibody response (42).

PncOMPC vaccine was given to 31 Finnish children at 24 months, and 10 of them also received it at 26 months. The primary response was only slightly higher than to the PS vaccine, but after the second dose a booster type response was seen in most of the vaccinees (43). Studies conducted during the second year of life showed that the heptavalent PncOMPC conjugate was more immunogenic than the PS vaccine (44,45). Different formulations of PncCRM have also been tested in toddlers (46). Conjugates were more immunogenic than the PS vaccine; furthermore, the PS conjugate was more immunogenic than the OS conjugate. One study showed a good booster response to PS vaccine after primary immunization with pentavalent PS-based PncCRM (47). The PncT and PncD conjugates have also proven immunogenic in toddlers. A Finnish study compared 3-µg and 10-µg doses at 24 months, and a U.S. study used 10-µg doses of type 19F conjugates with PS vaccine booster doses (48).

### Infants

Keyserling et al. (49) have compared different dosages of type 14 PS containing monovalent PncOMPC vaccine in infants and shown that 2.5µg to 5µg of type 14 PS in the conjugate gave better responses than the lower doses. A Finnish study (50) showed that a primary series of three doses of tetravalent PncOMPC at 2, 4, and 6 months was better than two doses at 4 and 6 months. Furthermore, a booster dose of PncOMPC given at 14 months evoked a secondary response to all PS types. Concomitant administration of PncOMPC with routine infant immunizations does not seem to have an effect on either anti-Hib or anti-Pnc PS antibody responses (51). The heptavalent PncOMPC formulation is as immunogenic as the previous formulations with fewer serotypes (27).

Åhman et al. have shown that the pentavalent PncCRM vaccine containing OS derived from pneumococcal capsule was immunogenic and tolerable in infants (52). The same children developed a good antibody response when boostered with PS vaccine at 24 months, suggesting that the immunologic priming had been good even if the antibody response to the primary series had remained rather low (53). The PS-based PncCRM has been shown to be more immunogenic than OS conjugates also in infancy (54). A Gambian study evaluated the pentavalent PncCRM conjugate (PS-based) in a developing country when given at 2, 3, and 4 or at 2 and 4 months. The vaccine was immunogenic and well tolerated; the schedule of three doses was better than the two-dose schedule (55).

A Finnish study compared three dosages of 1µg to 10µg of each PS in tetravalent PncT and PncD conjugates when administered at 2, 4, and 6 months. These vaccines were immunogenic in infancy, and no difference could be shown between PncT and PncD. The response after a primary series to PncD, but not to PncT, was dose dependent (56). The children immunized with PncD in infancy had a booster response after reimmunization with either PncD or pneumococcal PS vaccine at 14 months (57). All who received PncT were boostered with PS vaccine, and the response was dose dependent; children that had received 10-µg doses of PncT during the primary immunization had the lowest mean booster responses (58). Another Finnish study showed that octavalent (types 3, 4, 6B, 9V, 14, 18C, 19F, and 23F) PncD (3µg of each PS) and PncT (1µg of each PS) induced immune responses similar to the respective tetravalent formulations (29). An Icelandic study showed that the octavalent vaccine was immunogenic in infants when given at 3, 4, and 6 months and that the IgG anti-PS concentrations correlated with the opsonic activity (59).

Because no study has directly compared different pneumococcal conjugate vaccines,

Table 2. Antibody response of Finnish infants to pneumococcal conjugate vaccines administered at 2, 4, and 6 months of age*.

| | Geometric Mean of the Anti-PNC PS (µg/ml) | | | | | | | | |
|---|---|---|---|---|---|---|---|---|---|
| | Type 6B | | Type 14 | | Type 19F | | Type 23F | | |
| Vaccine | Pre | Post | Pre | Post | Pre | Post | Pre | Post | Ref. |
| PncOMPC | 0.17 | 1.30 | 0.42 | 8.27 | 0.34 | 9.85 | 0.28 | 1.90 | (50) |
| PncCRM | 0.25 | 0.50 | 0.30 | 2.49 | 0.46 | 1.13 | 0.18 | 0.83 | (52) |
| PncT01-4 | 0.25 | 0.89 | 0.24 | 2.84 | 0.36 | 3.73 | 0.18 | 0.82 | (56) |
| PncT01-8 | 0.20 | 1.28 | 0.30 | 2.56 | 0.56 | 4.23 | 0.22 | 1.03 | (29) |
| PncD03-4 | 0.26 | 0.88 | 0.44 | 2.20 | 0.43 | 5.29 | 0.21 | 0.67 | (56) |
| PncD03-8 | 0.17 | 1.44 | 0.31 | 4.62 | 0.37 | 4.94 | 0.24 | 1.07 | (29) |

PncOMP = tetravalent conjugate vaccine with a meningococcal outer membrane protein complex as a carrier
PncCRM = pentavalent oligosaccharide conjugate vaccine with CRM197 protein as a carrier
PncT01-4 = tetravalent conjugate vaccine with tetanus toxoid carrier; 1µg of each of four polysaccharides
PncT01-8 = tetravalent conjugate vaccine with tetanus toxoid carrier; 1µg of each of four polysaccharides
PncD03-4 = tetravalent conjugate vaccine with diphtheria toxoid carrier; 3µg of each of four polysaccharides
PncD03-8 = octavalent conjugate vaccine with diphtheria toxoid carrier; 3µg of each of four polysaccharides
*Serum samples are taken before immunization (pre) and at 7 months (post). The data have been gathered from separate studies done in the same population.

the comparison has to rely on data from separate studies. A Finnish group has analyzed the antibody response in adults, toddlers, and infants to all four types of Pnc conjugates. This comparison shows that there are vaccine- and type-specific differences in the antibody responses (Table 2). However, none of the vaccines used in these studies have the composition suggested in the phase-III trials (29,50,52,56).

Comparing the data from different studies is difficult because there can be interlaboratory variation in the enzyme-linked immunosorbent assay results. The Centers for Disease Control and Prevention, Food and Drug Administration, and World Health Organization are working on a standardized anti-Pnc PS assay, which will, if not eliminate, at least reduce the impact of this problem. A standard serum (60) to be used in all laboratories is distributed by Center for Biologics Evaluation and Research/Food and Drug Administration.

### Pneumococcal Conjugates and the Carriage of Pnc

Experience with the Hib conjugates (24) suggests that Pnc conjugate vaccines could also reduce the number of carriers of the vaccine types and in this way decrease the spread of bacteria. The results from the only reported study are encouraging. The PncOMPC vaccine decreased the carriage rate among toddlers, while the pneumococcal PS vaccine did not (45). Importantly, the carriage of antibiotic-resistant Pnc also decreased (61).

### Efficacy Studies

Phase-III studies with the heptavalent formulations of PncOMPC and PncCRM are ongoing or being started. These studies look at prevention of carriage, acute otitis media, or invasive Pnc infection caused by Pnc of the vaccine serotypes. Furthermore, there are several plans for studying the effect of Pnc conjugates on Pnc invasive infection and pneumonia in developing countries.

### Questions to Be Answered in the Future

We do not know if conjugate vaccines can really prevent Pnc infections better than the PS vaccine. We hope that the new vaccines can prevent several types of infections, from symptomless Pnc carriage to serious invasive infections with high death rates. It is quite probable that the protective immune response needed is different for each type of infection. We do not know if parenterally administered vaccine can prevent carriage or mucosal infections such as acute otitis media. It is still unknown whether a mucosal immune response is needed or whether transudation of antibodies from the serum is enough for protection against local infection. Saliva samples of infants immunized with Hib conjugate vaccines contain secretory IgA but also IgG, which has most probably transudated from serum (62). In an infant rat Hib colonization model, both secretory IgA and serum derived IgG decreased colonization (63). Furthermore, animal experiments suggest that immune response evoked by parenteral administration of a conjugate vaccine would alone protect against acute otitis media (64). In addition, passive immunization of infants with hyperimmune serum pool containing antibodies to pneumococcal PS-induced protection against pneumococcal acute otitis media suggests that protection is offered when high enough serum antibody concentrations are gained (65). At present, there are no data to show which antibody concentrations are needed for protection. Deciding about the protective concentration might be difficult because the development of immunologic memory is an important factor; the protection does not solely depend on the existing antibody concentration.

Most phase-II studies have used a schedule of two or three doses of Pnc conjugate vaccine in infancy (usually at 2, 4, and 6 months) and a booster dose of either conjugate or Pnc PS at the second year of life. The need for a booster dose at the second year is not known; this information would be important especially for planning the vaccination schedules for developing countries, where administering a booster dose can be problematic. The experience from the Hib conjugates suggests that a booster dose might not be needed; the United Kingdom has successfully used a schedule of three doses at 3, 4, and 5 months without a booster dose (66).

Reduction of pneumococcal infections among the elderly would probably increase the quality of their lives. The immunogenicity of pneumococcal PS vaccine in this age group has been satisfactory (67,68). An Hib conjugate (PRP-D) has proven more immunogenic than the Hib PS vaccine in the elderly (69). However, the immune response to PncCRM was not better than to the Pnc PS vaccine, and no booster response was seen (70). Studies with other pneumococcal conjugates in the elderly have not been reported.

Because pneumococcal infections of very young infants are a problem in developing countries, several groups have studied the possibility of maternal immunization with pneumococcal vaccines (71,72). So far only PS vaccines have been used; even though these vaccines are immunogenic in pregnant mothers, the immunity transferred to the neonate is not very long lasting. If the conjugate vaccines induce higher antibody concentrations in mothers, the concentration of passively acquired antibody in the baby would stay high for a longer time. The Hib conjugate vaccines induce good responses in mothers and, consequently, long-lasting protective concentrations in infants born to these mothers (73,74). The effect of simultaneous maternal tetanus immunization, especially if conjugates with a tetanus toxoid carrier are used, and the effect of high maternal antibody level on the antibody responses of the infants have to be determined.

By the year 2000, we may have pneumococcal conjugate vaccines to include in routine childhood immunization programs. The price of the conjugate vaccines has been so high that their use throughout the world has not been possible. An important challenge in developing of pneumococcal conjugate vaccines is to reduce the costs of manufacturing so that all children can benefit from them.

*Dr. Käyhty is a senior researcher at the National Public Health Institute, Finland, working as the head of the Laboratory of Vaccine Immunology. Her research focuses on systemic and mucosal immunity against encapsulated bacteria, mainly* Neisseria meningitidis, Streptococcus pneumoniae, *and* Haemophilus influenzae *type b.*

*Dr. Eskola is a research professor at the National Public Health Institute, Finland, working as the director of the Division of Infectious Diseases, and the head of the Department of Vaccines. A pediatric infectious disease physician by training, he has focused his research interests on the clinical evaluation of new vaccines. He is the principal investigator of the Finnish efficacy trial of pneumococcal conjugates in prevention of acute otitis media.*

### References

1. Alho OP. Acute otitis media in infancy: Occurrence and risk factors. Oulu, Finland: University of Oulu, 1990.

2. Luotonen J, Herva E, Karma P, Timonen M, Leinonen M, Mäkelä PH. The bacteriology of acute otitis media in children with special reference to *Streptococcus pneumoniae* as studied by bacteriological and antigen detection methods. Scand J Infect Dis 1981;13:177-83.
3. Stansfield SK. Acute respiratory infections in the developing world: strategies for prevention, treatment and control. Pediatr Infect Dis J 1987;6:622-9.
4. Eskola J, Takala AK, Kela E, Pekkanen E, Kalliokoski R, Leinonen M. Epidemiology of invasive pneumococcal infections in children in Finland; A five year prospective study with special implications for prevention. JAMA 1992;268:3323-7.
5. Dagan R, Englehard D, Piccard E, Israeli Pediatric Bacteremia and Meningitis Group. Epidemiology of invasive childhood pneumococcal infections in Israel. JAMA 1992;268:3328-32.
6. Bennett NM, Buffington J, LaForce FM. Pneumococcal bacteremia in Monroe County, New York. Am J Public Health 1992;82:1513-6.
7. Advisory Committee on Immunization Practices. Prevention of pneumococcal disease. Recommendations of the Advisory Committee on Immunization Practices. MMWR (in press) 1996.
8. Klugman KP. Pneumococcal resistance to antibiotics. Clin Microbiol Rev 1990;3:171-96.
9. Sniadack DH, Schwartz B, Lipman H, Bogaerts J, Butler JC, Dagan R, et al. Potential interventions for the prevention of childhood pneumonia: geographic and temporal differences in serotype and serogroup distribution of sterile site pneumococcal isolates from children- implications for vaccine strategies. Pediatr Infect Dis J 1995;14:503-10.
10. Shapiro ED, Berg AT, Austrian R, Schroeder D, Parcells V, Margolis A, et al. The protective efficacy of polyvalent pneumococcal polysaccharide vaccine. N Engl J Med 1991;325:1453.
11. Butler JC, Breiman RF, Campbell JF, Lipman HB, Broome CV, Facklam RR. Pneumococcal polysaccharide vaccine efficacy. JAMA 1993;270:1826-31.
12. Riley ID, Lehmann D, Alpers MP, Marshall TF, Gratten H, Smith D, et al. Pneumococcal vaccine prevents death from acute lower respiratory tract infections in Papua New Guinean children. Lancet 1986;2:877-81.
13. Mäkelä PH, Sibakov M, Herva E, Henricksen J. Pneumococcal vaccine and otitis media. Lancet 1980;2:547-51.
14. Käyhty H, Eskola J, Peltola H, Stout M, Samuelson JS, Gordon LK. Immunogenicity in infants of a vaccine composed of *Haemophilus influenzae* type b capsular polysaccharide mixed with DPT or conjugated to diphtheria toxoid. J Infect Dis 1987;155:100-6.
15. Sarvas H, Rautonen N, Sipinen S, Mäkelä O. IgG subclasses of pneumococcal antibodies—effect of allotype G2m(n). Scand J Immunol 1989;29:229-37.
16. Mäkelä O, Mattila P, Rautonen N, Seppälä N, Seppälä I, Eskola J, et al. Isotype concentrations of human antibodies to *Haemophilus influenzae* type b polysaccharide (Hib) in young adults immunized with the polysaccharide as such or conjugated to a protein (diphtheria toxoid). J Immunol 1987;139:1999-2004.
17. Lock RA, Paton JC, Hansman D. Comparative efficacy of pneumococcal neuraminidase and pneumolysin as immunogens protective against *Streptococcus pneumoniae*. Microb Pathog 1988;5:461-7.
18. Tart RC, McDaniel LS, Ralph BA, Briles DE. Truncated *Streptococcus pneumoniae* PspA molecules elicit cross-protective immunity against pneumococcal challenge in mice. J Infect Dis 1996;173:380-6.
19. Sampson JS, O'Connor SP, Stinson AR, Tharpe JA, Russell H. Cloning and nucleotide sequence analysis of psaA, the *Streptococcus pneumoniae* gene encoding a 37-kilodalton protein homologous to previously reported *Streptococcus sp.* adhesins. Infect Immun 1994;62:319-24.
20. Alexander JE, Lock RA, Peeters C, Poolman JT Andrew PW, Mitchell TJ, et al. Immunization of mice with pneumolysin toxoid confers a significant degree of protection against at least nine serotypes of *Streptococcus pneumoniae.* Infect Immun 1994;62:5683-8.
21. Paton JC, Lock RA, Hansman DJ. Effect of immunization with pneumolysin on survival time of mice challenged with *Streptococcus pneumoniae.* Infect Immun 1983;40:548-52.
22. McDaneiel LS, Sheffield JS, Delucchi P, Briles DE. PspA, a surface protein of *Streptococcus pneumoniae*, is capable of eliciting protection against pneumococci of more than one capsular type. Infect Immun 1991;59:222-8.
23. Goebel W, Avery OT. Chemo-immunological studies on conjugated carbohydrate proteins. I. The synthesis of p-aminophenol ß-glucoside, p-aminophenol ß-galactoside and their coupling with serum globulin. J Exp Med 1929;50:521-33.
24. Mäkelä PH, Eskola J, Käyhty H, Takala A. Vaccines against *Haemophilus influenzae* type b. In: Ala-Aldeen D, Hormaeche C, editors. Molecular and Clinical Aspects of Bacterial Vaccine Development. Chichester: John Wiley & Sons, Ltd., 1995:41-91.
25. Eskola J, Käyhty H, Takala AK, Peltola H, Rönnberg P-R, Kela E, et al. A randomized, prospective field trial of a conjugate vaccine in the protection of infants and young children against invasive *Haemophilus influenzae* type b disease. N Engl J Med 1990;323:1381-7.
26. Barbour ML, Booy R, Crook DW, Griffiths H, Chapel HM, Moxon ER, et al. *Haemophilus influenzae* type b carriage and immunity four years after receiving the *Haemophilus influenzae* oligosaccharide-CRM197 (HbOC) conjugate vaccine. Pediatr Infect Dis J 1993;12:478-84.

27. Anderson EL, Kennedy DJ, Geldmacher KM, Donnelly J, Mendelman PM. Immunogenicity of heptavalent pneumococcal conjugate vaccine in infants. Pediatrics 1996;128:649-53.
28. Hogerman D, Kimura A, Malinoski F, Treanor J. Safety and immunogenicity of a heptavalent pneumococcal conjugate vaccine in healthy adult volunteers. Presented at the Infectious Diseases Society of America, Annual Meeting, San Francisco, CA, 1995; Abstract #389, page 114.
29. Åhman H, Käyhty H, Leroy O, Froeschle J, Eskola J. Immunogenicity of octavalent pneumococcal (Pnc) conjugate vaccines (PncD, PncT) in Finnish infants. 36th Interscience Conference on Antimicrobial Agents and Chemotherapy (ICAAC), New Orleans, LA, 1996; Abstract G040, page 150.
30. Lee C-J, Lock RA, Andrew PW, Mitchell TJ, Hansman D, Paton JC. Protection of infant mice from challenge with *Streptococcus pneumoniae* type 19F by immunization with a type 19F polysaccharide-pneumolysoid conjugate. Vaccine 1994;12:785-878.
31. Schneerson R, Levi L, Robbins JB, Bryla DM, Schiffman G, Lagergard T. Synthesis of a conjugate vaccine composed of pneumococcus type 14 capsular polysaccharide bound to pertussis toxin. Infect Immun 1992;60:3528-32.
32. van de Wijgert JHHM, Verheul AFM, Snippe H, Check IJ, Hunter RL. Immunogenicity of *Streptococcus pneumoniae* type 14 capsular polysaccharide: influence of carriers and adjuvants on isotype distribution. Infect Immun 1991;59:2750-7.
33. Alonso de Velasco E, Merkus D, Anderton S, Verheul AFM, Lizzio EF, van der Zee R, et al. Synthetic peptides representing T-cell epitopes act as carriers in pneumococcal polysaccharide conjugate vaccines. Infect Immun 1995;63:961-8.
34. Schneerson R, Robbins JB, Parke JC, Bell C, Schlesselman JJ, Sutton A, et al. Quantitative and qualitative analyses of serum antibodies elicited in adults by *Haemophilus influenzae* type b and pneumococcus type 6A capsular polysaccharide-tetanus toxoid conjugates. Infect Immun 1986;52:519-28.
35. Vella PP, Marburg S, Staub JM, Kniskern PJ, Miller W, Hagopian A, et al. Immunogenicity on conjugate vaccines consisting of pneumococcal capsular polysaccharide type 6B, 14, 19F, and 23F and meningococcal outer membrane protein complex. Infect Immun 1992;60:4977-83.
36. Fattom A, Vann WF, Szu SC, Sutton A, Li X, Bryla D, et al. Synthesis and physicochemical and immunological characterization of pneumococcus type 12F polysaccharide-diphtheria toxoid conjugates. Infect Immun 1988;56:2292-8.
37. Giebink GS, Koskela M, Vella PP, Harris M, Le CT. Pneumococcal capsular polysaccharide-meningococcal outer membrane protein complex conjugate vaccines; immunogenicity and efficacy in experimental pneumococcal otitis media. J Infect Dis 1993;167:347-55.
38. Kennedy EL, Anderson EL. Safety and immunogenicity of a heptavalent pneumococcal conjugate vaccine in adults and children. Presented at the 33rd Interscience Conference on Antimicrobial Agents and Chemotherapy (ICAAC), New Orleans, LA, 1993; Abstract #167, page 150.
39. Nieminen T, Virolainen A, Käyhty H, Leinonen M, Eskola J. Immune response to tetravalent pneumococcal conjugate vaccine in adults. Presented at the 32nd Interscience Conference on Antimicrobial Agents and Chemotherapy (ICAAC), Anaheim, CA, 1992; Abstract #1283, page 324.
40. Malinoski F, Hogerman D, Ginsberg H, Madore D. Safety and immunogenicity of pentavalent *S. pneumoniae* conjugate vaccines in healthy adults. Presented at the 33rd Interscience Conference on Antimicrobial Agents and Chemotherapy (ICAAC), New Orleans, LA, 1993; Abstract #168, page 150.
41. Portier H, Choutet P, Duong M, Moreau M, Danve B. Serum antibody response to a tetravalent pneumococcal-tetanus toxoid conjugate vaccine in adult volunteers. Presented at the 34th Interscience Conference on Antimicrobial Agents and Chemotherapy (ICAAC), Orlando, FL, 1994; Abstract #G91, page 237.
42. Nieminen T, Käyhty H, Eskola J. Mucosal and serum immune response to tetravalent pneumococcal conjugate vaccines in adults. Presented at the 34th Interscience Conference on Antimicrobial Agents and Chemotherapy (ICAAC), Orlando, FL, 1994; Abstract #G89, page 237.
43. Käyhty H, Rönnberg P-R, Virolainen A, Eskola J. Immunogenicity of tetravalent pneumococcal capsular polysaccharide-meningococcal outer membrane protein conjugate vaccine in Finnish 2-year old children. Presented at the 33rd Interscience Conference on Antimicrobial Agents and Chemotherapy (ICAAC), New Orleans, LA, 1993; Abstract #172, page 151.
44. Zangwill K, Melamed R, Ward J, Marcy S, Patridge S, Greenberg D, et al. Safety and immunogenicity of a heptavalent pneumococcal conjugate vaccine among children 12-24 months of age. Presented at the Annual Meeting of the American Pediatric Society/ Society for Pediatric Research, San Diego, CA, 1995; Abstract #1130, page 191A.
45. Dagan R, Melamed R, Abramson O, Piglansky L, Greenberg D, Mendelman P, et al. Effect of heptavalent pneumococcal-OMPC conjugate vaccine on nasopharyngeal carriage when administered during the 2nd year of life. Presented at the Annual Meeting of the American Pediatric Society/Society for Pediatric Research, San Diego, CA, 1995; Abstract #1020, page 172A.
46. Steinhoff D, Edward K, Keyseling H, Thoms ML, Johnson C, Madore D, et al. A randomized comparison of three bivalent *Streptococcus pneumoniae* glycoprotein conjugate vaccines in young children: effect of polysaccharide size and linkage characteristics. Pediatr Infect Dis J 1994;13:368-72.

47. Chiu SS, Grenberg DP, Partride S, et al. Safety and immunogenicity of a pentavalent pneumococcal conjugate vaccine in healthy toddlers. Presented at the 35th Interscience Conference on Antimicrobial Agents and Chemotherapy (ICAAC), San Francisco, CA, 1995; Abstract #G71, page 171.
48. Kennedy D, DeRousse C, Anderson E. Immunologic response of 12-18 month old children to licensed pneumococcal polysaccharide vaccine primed with *Streptococcus pneumoniae* 19F conjugate vaccine. Presented at the 34th Interscience Conference on Antimicrobial Agents and Chemotherapy (ICAAC), Orlando,FL, 1994; Abstract #G88, page 236.
49. Keyserling H, Bosley C, Starr S, Watson B, Laufer D, Anderson E, et al. Immunogenicity of pneumococcal type 14 conjugate vaccine in infants. Presented at the Annual Meeting of the American Pediatric Society/ Society for Pediatric Research, Seattle, WA, 1994; Abstract #1087, page 184A.
50. Käyhty H, Åhman H, Rönnberg P-R, Tillikainen R, Eskola J. Pneumococcal polysaccharide-meningococcal outer membrane protein complex conjugate vaccine is immunogenic in infants. J Infect Dis 1995;172:1273-8.
51. Yogev R, Gupta S, Emanuel B, William K, Adams J. Safety, tolerability and immunogenicity of tetravalent (6B, 14, 19F, 23F) pneumococcal (Pn) conjugate vaccine in infants given concurrently with routine immunizations. Presented at the 33rd Interscience Conference on Antimicrobial Agents and Chemotherapy (ICAAC), New Orleans, LA, 1993; Abstract #170, page 150.
52. Åhman H, Käyhty H, Tamminen P, Uistola A, Malinoski F, Eskola J. Pentavalent pneumococcal oligosaccharide conjugate vaccine PncCRM is well tolerated and able to induce an antibody response in infants. Pediatr Infect Dis J 1996;15:134-9.
53. Käyhty H, Åhman H, Vuorela A, Malinkoski F, Eskola J. Response at 24 months to a booster dose to pneumococcal (Pnc) polysaccharide (PS) vaccine in children immunized with pentavalent Pnc conjugate vaccine (PncCRM) in infancy. Presented at the 36th Interscience Conference on Antimicrobial Agents and Chemotherapy (ICAAC), New Orleans, LA, 1996; Abstract #G108, page 162.
54. Daum RS, Steinhoff M, Rennels M, Rothstein E, Resinger K, Keyserling H, et al. Immunogenicity of *S. pneumoniae* oligo- and polysaccharide-CRM197 conjugate vaccines in healthy US infants. Presented at the 35th Interscience Conference on Antimicrobial Agents and Chemotherapy (ICAAC), San Francisco, CA, 1995; Abstract #GG5, page 170.
55. Leach A, Ceesay SJ, Banya WAS, Greenwood BM. Pilot trial of a pentavalent pneumococcal polysaccharide/protein conjugate vaccine in Gambian infants. Pediatr Infect Dis J 1996;15:333-9.
56. Åhman H, Käyhty H, Leroy O, Froeschle J, Eskola J. Immunogenicity of tetravalent pneumococcal conjugate vaccines (PncD, PncT) in Finnish infants. Presented at the 35th Interscience Conference on Antimicrobial Agents and Chemotherapy (ICAAC), San Francisco, CA, 1995; Abstract #G69, page 170.
57. Åhman H, Käyhty H, Leroy O, Froeschle J, Eskola J. Booster response to polysaccharide and conjugate vaccine at 14 months after immunization with tetravalent pneumococcal (Pnc) conjugate vaccine PncD in infancy. Presented at the 36th Interscience Conference on Antimicrobial Agents and Chemotherapy (ICAAC), New Orleans, LA, 1996; Abstract G110, page 163.
58. Åhman H, Käyhty H, Leroy O, Eskola J. Booster response to polysaccharide vaccine at 14 months after immunization with tetravalent pneumococcal (Pnc) conjugate vaccine PncT in infancy is dose dependent. Presented at the 36th Interscience Conference on Antimicrobial Agents and Chemotherapy (ICAAC), New Orleans, LA, 1996; Abstract #G109, page 162.
59. Jonsdottir I, Sigurdardottir STH, Vidarsson G, Ingolfsdottir G, Gudnason T, Dadidsdottir K, et al. Pneumococcal conjugate vaccines elicit functional antibodies in infants. Scand J Immunol 1996;43:710.
60. Quataert SA, Kirch CS, Quackenbush Wiedl LJ, Phipps DC, Strohmeyer S, Cimino CO, et al. Assignment of weight-based antibody units to a human antipneumococcal standard reference serum, Lot 89-S. Clin Diagn Lab Immunol 1995;2:590-7.
61. Dagan R, Melamed R, Muallem M, Piglansky L, Greenberg D, Abramson O, et al. Reduction of nasopharyngeal carriage of penicillin-resistant pneumococci by pneumococcal-OMPC conjugate vaccine during second year of life. Presented at the 35th Interscience Conference on Antimicrobial Agents and Chemotherapy (ICAAC), San Francisco, CA, 1995; Abstract #2, page 158.
62. Kauppi M, Eskola J, Käyhty H. Anti-capsular polysaccharide antibody concentrations in saliva after immunization with *Haemophilus influenzae* type b conjugate vaccines. Pediatr Infect Dis J 1995;14:286-94.
63. Kauppi M, Saarinen L, Käyhty H. Anti-capsular polysaccharide antibodies reduce nasopharyngeal colonization by *Haemophilus influenzae* type b in infant rats. J Infect Dis 1993;167:365-71.
64. Giebink GS, Meier JD, Quartey MK, Liebeler CL, Le CT. Immunogenicity and efficacy of *Streptococcus pneumoniae* polysaccharide-protein conjugate vaccines against homologous and heterologous serotypes in the chinchilla otitis media model. J Infect Dis 1996;173:119-27.
65. Shurin PA, Rehmus JM, Johnson CE, Marchant CD, Carlin SA, Super DM, et al. Bacterial polysaccharide immune globulin for prophylaxis of acute otitis media in high-risk children. J Pediatr 1993;123:801-10.
66. Booy R, Hodgson S, Carpenter L, Mayon-White RT, Slack MPE, Macfarlane JA, et al. Efficacy of *Haemophilus influenzae* type b conjugate vaccine PRP-T. Lancet 1994;344:362-6.
67. Sankilampi U, Honkanen PO, Bloigu A, Herva E, Leinonen M. Antibody response to pneumococcal capsular polysaccharide vaccine in the elderly. J Infect Dis 1996;173:387-93.

68. Musher DM, Groover JE, Graviss A, Baughn RE. The lack of association between aging and postvaccination levels of IgG antibody to capsular polysaccharide of *Streptococcus pneumoniae*. Clin Infect Dis 1996;22:165-7.
69. Powers D, Moore S, Mink CM. Vaccination of elderly adults with *Haemophilus influenzae* type b (Hib) polysaccharide or conjugate vaccine. Presented at the 35th Interscience Conference on Antimicrobial Agents and Chemotherapy (ICAAC), San Francisco, CA, 1995; Abstract #976, page 171.
70. Powers DC, Anderson EL, Lottenbach K, Mink CM. Reactogenicity and immunogenicity of a protein-conjugated pneumococcal oligosaccharide vaccine in older adults. J Infect Dis 1996;173:1014-8.
71. Shahid NS, Steinhoff MC, Hoque SS, Begum T, Thompson C, Siber GR. Serum, breast milk, and infant antibody after maternal immunisation with pneumococcal vaccine. Lancet 1995;346:1252-7.
72. O'Dempsey TJD, McArdle T, Ceesay S, Banya WAS, Demba E, Secka O, et al. Immunization with a pneumococcal capsular polysaccharide vaccine during pregnancy. Vaccine (in press).
73. Englund JA, Glezen WP, Turner C, Harvey J, Thompson C, Siber GR. Transplacental antibody transfer following maternal immunization with polysaccharide and conjugate *Haemophilus influenzae* type b vaccines. J Infect Dis 1995;171:99-105.
74. Mulholland K, Rahaman O, Suara R, Siber G, Roberton D, Jaffar S, et al. Maternal immunization with *Haemophilus influenzae* type b polysaccharide-tetanus protein conjugate vaccine in the Gambia. JAMA 1996;275:1182-8.
